# Impact of Mycorrhiza Inoculations and Iron Amino Chelate on Growth and Physiological Changes of Cucumber Seedlings Across Different pH Levels

**DOI:** 10.3390/plants14030341

**Published:** 2025-01-23

**Authors:** Saber Mohammadnia, Maryam Haghighi, Maryam Mozafarian, András Geösel

**Affiliations:** 1 Department of Horticulture, College of Agriculture, Isfahan University of Technology, Isfahan 84156-83111, Iran; 2 Department of Vegetable and Mushroom Growing, Hungarian University of Agriculture and Life Sciences, 1118 Budapest, Hungary; geosel.andras@uni-mate.hu

**Keywords:** amino chelate, arbuscular mycorrhizal fungi, chlorophyll, cucumber (*Cucumis sativus* L.), Fe sources

## Abstract

Cucumber, a vital greenhouse crop, thrives in soils with a pH range of 5.5–6.5, yet the combined effects of arbuscular mycorrhizal fungi (AMF) and iron amino chelates on its growth and physiological responses across varying pH levels remain underexplored. This study used a factorial design in a completely randomized setup with three replications and was conducted at the Horticulture Department of Isfahan University of Technology. The aim of this study was to investigate the effects of AMF inoculation (*Glomus mosseae*) and iron amino chelates on the growth and physiological responses of cucumber plants at various pH levels. Treatments included two levels of AMF inoculation (non-inoculated as m1 and inoculated as m^2^), three levels of iron concentration (f1: no iron, f2: Johnson’s nutrient solution, f3: Johnson’s solution with iron amino chelate), and three pH levels (pH 5 (p1), pH 7 (p2), and pH 8 (p3)). The moisture was maintained at field capacity throughout the study. The results demonstrated that mycorrhizal inoculation at pH 7 significantly improved key traits, including chlorophyll content, photosynthesis rate, stomatal conductance, phenol content, and antioxidant activity. Mycorrhizal inoculation combined with 2 ppm of Fe amino chelate at pH 7 led to the highest improvement in shoot fresh weight of cucumber and physiological traits. However, at pH 7 without mycorrhiza, stress indicators such as ABA levels and antioxidant enzyme activities (SOD, POD, CAT, and APX) increased, highlighting the protective role of AMF under neutral pH conditions. In contrast, pH 5 was most effective for enhancing root and stem fresh weight. The lower pH may have facilitated better nutrient solubility and uptake, promoting root development and overall plant health by optimizing the availability of essential nutrients and reducing competition for resources under more acidic conditions. These findings highlight the potential of combining mycorrhizal inoculation with iron amino chelates at pH 7 not only to enhance cucumber growth and resilience in nutrient-limited environments but also to contribute to sustainable agricultural practices that address global challenges in food security and soil health.

## 1. Introduction

Cucumber (*Cucumis sativus* L.), a species belonging to the Cucurbitaceae family, is widely grown in greenhouse environments [1]. Globally, it ranks among the most important vegetables, behind tomato, cabbage, and onion [2], and is mainly cultivated during the summer months [3]. Asia contributes 90.1% of the world’s total cucumber production [4]. In addition to its nutritional value, cucumber is a good source of vital vitamins and minerals, such as calcium, potassium, silica, phosphorus, and magnesium [3].

Iron (Fe) is an essential micronutrient for plant development and plays a critical role in numerous physiological and metabolic functions, including photosynthesis, respiration, electron transport, nitrogen fixation, and nitrate reduction [5]. Fe also plays a significant role in plant enzymatic antioxidant defense systems [6]. Despite its importance, Fe deficiency is a common nutritional issue, particularly in plants grown in alkaline and calcareous soils [7]. This deficiency triggers oxidative stress in cells, leading to reduced photosynthetic pigment production and chlorosis [8]. Additionally, a lack of Fe disrupts the formation of protein–membrane complexes, causing their breakdown, reducing chloroplast lamella, and eventually leading to chloroplast collapse, which negatively affects plant growth, resistance, fruit yield, and quality [9,10].

Fertilizers are vital for nutrient supply to soil, supporting plant growth, and improving crop yields [11]. Common Fe fertilizers used in agriculture include chelated iron, inorganic iron, and organic iron sources [12]. When these soluble fertilizers are added to soil, processes such as exchange/sorption, complexation, precipitation, and dissolution occur. These interactions are influenced by soil elements like iron oxides, clay, and calcium, which in turn impact the effectiveness of fertilizers in supporting plant growth [13].

Chelating agents are widely used in agriculture to improve the bioavailability of essential nutrients like Fe and zinc (Zn). Common chelating agents include EDTA and EDDHA, which are effective but raise environmental concerns due to their potential as pollutants [14]. In contrast, amino-chelated modern formulations based on amino acids offer a more sustainable alternative. These chelates enhance nutrient uptake with fewer side effects and less environmental impact [15]. Recent studies have demonstrated the application of iron chelates in various crops, including tomato [16] and cucumber [17], highlighting their effectiveness in improving plant health. Amino chelates bind tightly to metal ions, improving their absorption and transport within the plant. Iron-chelated nano-fertilizers, for instance, offer stable and controlled Fe release, making them particularly useful in saline-alkali soils where Fe tends to precipitate and become unavailable [18]. In addition to their role in nutrient absorption, amino chelates contribute to protein synthesis, hormone production, and the biosynthesis of important metabolites like chlorophyll, improving overall plant health and growth [19,20].

Recent research highlights the potential of using specific soil microorganisms as a sustainable and eco-friendly approach to alleviating Fe deficiency in plants [21,22]. Arbuscular mycorrhizal fungi (AMF), mainly from the Glomeromycota phylum, form symbiotic relationships with vascular plants, enhancing nutrient uptake through their extensive hyphal networks, especially under nutrient-deficient conditions [23]. AMF symbiosis activates or maintains nutrient transporter mechanisms in plants, and the regulation of gene expression can induce both cytological and metabolic changes [23]. Approximately 85% of plant species in terrestrial ecosystems engage in mutualistic relationships with AMF [24].

Liu et al. [25] demonstrated that root arbuscular mycorrhizae in cucumber significantly increase leaf osmolyte levels, ensuring that mycorrhizal plants maintain effective osmotic regulation under stress conditions. These fungi create structures like arbuscules, vesicles, and hyphal coils within plant roots, using their hyphal network to gather nutrients such as phosphorus, nitrogen, potassium, and micronutrients from the soil, which they transfer to the host plant in exchange for carbohydrates [24,26]. This network significantly increases the absorption capacity of roots, extending beyond the nutrient depletion zone, thereby improving nutrient availability for plants.

Furthermore, the results of Xiang et al. [27] showed that AMF-inoculated cucumber plants exhibited greater resistance to stress conditions than uninoculated plants, which was closely linked to increased osmolyte content and the expression of stress-responsive genes (e.g., CsPIPs and CsHsp70). Therefore, mycorrhization of cucumber, particularly during the seedling stage, enhances growth and stress resistance. AMF are particularly effective in enhancing the uptake of micronutrients like copper (Cu), zinc (Zn), and iron, which are often limited in bioavailability due to their tendency to form insoluble compounds or their uneven distribution in soil [28]. Moreover, AMF can mobilize and solubilize micronutrients by secreting organic acids and enzymes, converting these elements into more plant-accessible forms [29]. By regulating micronutrient levels in plant tissues, AMF acts as a buffer, helping to prevent deficiencies or toxicities [30]. As a result, AMF plays a crucial role in improving nutrient uptake, converting less-available micronutrients into bioavailable forms, and maintaining nutrient balance in plants.

Despite extensive research on the individual benefits of AMF and chelating agents in improving nutrient uptake, a notable lack of comprehensive studies has examined their combined effects, particularly under varying pH levels in cucumber crops. This study aimed to address this gap by investigating the synergistic impact of mycorrhizal inoculation and iron amino chelate supplementation on iron absorption, plant growth, and the physiological responses of cucumbers under different pH conditions.

## 2. Results

The results of the analysis of variance (ANOVA) are detailed in Supplementary Tables S1–S4. The main effects of the treatments on the studied traits are presented in Supplementary Tables S5–S8.

### 2.1. The Interaction of AMF and pH Levels on Characteristics of the Seedling Cucumber

The interaction between AMF and pH was not significant for root volume, shoot diameter, and root dry weight. The highest shoot fresh weight was observed during AMF inoculation at pH 5 and pH 7. In contrast, no significant difference in shoot biomass was observed between the non-inoculated treatments at pH 5 and pH 8, with both showing greater fresh weight than at pH 8. In the absence of AMF inoculation, root fresh weight did not differ significantly among the treatments, all exhibiting high levels. However, during AMF inoculation, treatments at pH 5 and pH 7 produced significantly higher root fresh weight than those at pH 8 (*p* < 0.05). No significant difference was found between treatments at pH 5 and pH 7 during AMF inoculation, as both showed higher colonization rates than at pH 8 (Table 1).

The highest soil plant analysis development (SPAD) values were recorded at pH 5 and pH 7 with AMF inoculation, with no significant difference compared to the non-inoculated treatment at pH 5. Photosynthesis rate, transpiration, and stomatal conductance were significantly increased in plants inoculated with AMF at pH 7 (*p* < 0.05). The highest photosynthesis water use efficiency was recorded in AMF-inoculated plants at pH 5, whereas the highest mesophyll conductance was found in non-inoculated plants at pH 5 (Table 2).

AMF inoculation significantly increased the phenolic content of shoots, with the highest value observed at pH 7 (*p* < 0.05). During AMF inoculation, diphenyl-1-picrylhydrazyl radical (DPPH) percentages did not significantly differ across pH levels, remaining high. In addition, in the non-inoculated treatments, DPPH percentages did not differ significantly across pH levels (*p* < 0.05). Under non-inoculated conditions, the highest iron concentrations were observed at pH 5 and pH 8, which were statistically similar to the non-inoculated plants at pH 5 (*p* < 0.05). The highest abscisic acid (ABA) concentration was noted in non-inoculated plants at pH 5 and pH 8 compared with the other treatments (*p* < 0.05), with no significant differences among the other treatments, which all exhibited low levels. The highest activities of ascorbate peroxidase (APX), superoxide dismutase (SOD), and peroxidase (POX) enzymes were observed in non-inoculated plants at pH 5 and pH 8 compared with the other conditions (*p* < 0.05). No significant differences were observed among treatments for catalase (CAT) activity (Table 3).

### 2.2. The Interaction of AMF and Fe Concentrations on Characteristics of the Seedling Cucumber

The interaction between AMF and Fe was not significant for growth parameters. However, the highest percentage of root colonization was observed in the presence of 2 ppm Fe amino chelate compared with the other Fe concentrations (Table 4).

SPAD values during AMF inoculation were highest in the presence of 2 ppm Fe and Fe amino chelate compared with the other treatments. In plants inoculated with AMF and treated with 2 ppm of Fe amino chelate, the highest photosynthesis rate, transpiration, and stomatal conductance were recorded (*p* < 0.05). Additionally, the highest photosynthesis water use efficiency was found in inoculated plants treated with 2 ppm of Fe (*p* < 0.05) (Table 5).

Among treatments, the highest phenolic content was found in AMF-inoculated plants treated with 2 ppm amino chelate iron, followed by 2 ppm iron, which was 90% and 52% higher than the control (m2 f1), respectively (*p* < 0.05). AMF-inoculated plants in treatments with Fe 0 concentration and 2 ppm of Fe amino chelate exhibited higher DPPH levels compared to non-inoculation treatments (*p* < 0.05). Generally, the highest iron concentrations were observed in the non-inoculated treatment and in treatments with 2 ppm of Fe and 2 ppm of Fe amino chelate, as well as in the AMF-inoculated treatment with 2 ppm of Fe compared to others. The overall antioxidant activity was higher in non-inoculated plants than in inoculated plants, with the highest levels of ABA, APX, SOD, and POX observed in non-inoculated plants treated with 2 ppm of Fe and 2 ppm of Fe amino chelate (*p* < 0.05). No significant differences in CAT activity were noted among treatments (Table 6).

### 2.3. The Interaction of Level of pH and Fe Concentrations on Characteristics of the Seedling Cucumber

Root fresh weight at pH 5 did not differ significantly among iron treatments, with all treatments exhibiting high levels. Similarly, no significant differences were found between the iron levels at pH 7 and pH 8. The shoot dry weight was significantly increased by the application of 2 ppm of Fe at pH 5 compared with the control (*p* < 0.05) (Table 7).

SPAD values at both pH 5 and pH 7 were highest in the presence of 2 ppm of Fe and 2 ppm of Fe amino chelate compared with the other treatments (*p* < 0.05). Photosynthesis rates, stomatal conductance, and mesophyll conductance were highest at pH 5 in the Fe amino chelate compared to others (*p* < 0.05). The transpiration rates were higher in treatments with 2 ppm of Fe and 2 ppm of Fe amino chelate at pH 5 and pH 7, respectively (*p* < 0.05). The highest photosynthesis water use efficiency was recorded for 2 ppm Fe at pH 5 (Table 8).

Among the treatments, the highest phenolic content was observed in plants at pH 7 in the presence of 2 ppm amino chelate iron compared to others (*p* < 0.05). The iron concentrations in treatments at pH 5 and pH 8 were highest after the application of a Fe 0 concentration and 2 ppm of Fe, respectively, compared with the other treatments (*p* < 0.05). The highest ABA concentration was found in plants without Fe at pH 5, whereas enzyme activities peaked in the presence of a combination of pH 8 and 2 ppm of Fe (Table 9).

**Table 1:** Interactive effect of AMF and level of pH on growth characteristics of the cucumber.

Treatments		Root Volume (mL)	Shoot Fresh Weight (g per Plant)	Root Fresh Weight (g per Plant)	Shoot Diameter (mm)	Shoot Length (cm)	Shoot Dry Weight (g per Plant)	Root Dry Weight (g per Plant)	Root Colonization (%)
m1	p1	4.10 ± 0.93 a	396.04 ± 20.43 a	134.56 ± 14.05 a	5.64 ± 0.22 a	76.10 ± 3.19 a	36.40 ± 5.77 a	3.90 ± 1.81 a	0 ± 0.00 c
	p2	4.02 ± 1.08 a	296.64 ± 23.93 bc	95.20 ± 11.14 ab	5.12 ± 0.85 a	75.72 ± 3.91 a	32.63 ± 6.48 a	3.48 ± 1.62 a	0 ± 0.00 c
	p3	3.63 ± 0.86 a	329.62 ± 25.73 abc	114.18 ± 15.32 ab	5.32 ± 0.38 a	70.75 ± 2.86 b	30.17 ± 5.65 ab	3.78 ± 0.81 a	0 ± 00 c
m2	p1	4.80 ± 1.98 a	405.38 ± 22.94 a	142.49 ± 12.31 a	5.39 ± 0.16 a	74.14 ± 5.60 ab	34.68 ± 9.23 a	3.48 ± 1.97 a	75 ± 4.68 a
	p2	4.02 ± 0.74 a	353.73 ± 23.05 ab	106.24 ± 11.60 ab	5.66 ± 0.46 a	75.65 ± 4.96 a	33.75 ± 6.36 a	3.56 ± 0.65 a	80 ± 4.70 a
	p3	3.86 ± 1.57 a	257.60 ± 21.53 c	81.20 ± 10.24 b	5.43 ± 1.00 a	75.06 ± 3.53 a	25.51 ±7.97 b	3.48 ± 1.45 a	60 ± 4.70 b

Within a column, means followed by the same letter are not significantly different at *p* < 0.05 according to the least significant different test. Treatment includes non-inoculation with mycorrhiza (m1) and inoculation with mycorrhiza (m2). pH1 = 5 (p1), pH2 = 7 (p2), and pH3 =8 (p3).

**Table 2:** Interactive effect of AMF and level of pH on photosynthetic characteristics of the cucumber.

Treatments		Chlorophyll Index (SPAD Value)	Photosynthesis Rate (μmol (CO_2_) m^2^s^−1^)	Transpiration (mmol (H_2_O)m^2^s^−1^)	Stomata Conductance (mmol (H_2_O) m^2^ s^−1^)	Mesophyll Conductance (µmol)	Photosynthetic Water Use Efficiency (µM mol CO_2_ mol^−1^ H_2_O)
m1	p1	24.60 ± 3.91 ab	5.25 ± 0.32 d	5.76 ± 0.52 f	0.15 ± 0.02 f	0.56 ± 0.06 a	47.97 ± 4.81 b
	p2	23.29 ± 5.22 b	3.77 ± 1.70 f	6.09 ± 1.60 e	0.21 ± 0.04 e	0.01 ± 0.002 e	24.41 ± 2.79 e
	p3	19.34 ± 4.89 b	5.70 ± 0.73 c	7.24 ± 1.42 b	0.27 ± 0.09 b	0.01 ± 0.002 d	31.16 ± 3.22 d
m2	p1	31.92 ± 5.36 a	7.83 ± 1.23 b	6.82 ± 1.30 d	0.24 ± 0.16 c	0.02 ± 0.002 c	52.65 ± 4.45 a
	p2	32.07 ± 5.64 a	7.90 ± 1.52 a	9.17 ± 2.75 a	0.30 ± 0.08 a	0.05 ± 0.003 b	43.61 ± 4.02 c
	p3	20.66 ± 4.01 b	3.88 ± 0.16 e	7.12 ± 2.26 c	0.22 ± 0.06 d	0.01 ± 0.002 e	23.80 ± 1.44 f

Within a column, means followed by the same letter are not significantly different at *p* < 0.05 according to the least significant different test. Treatment includes non-inoculation with mycorrhiza (m1) and inoculation with mycorrhiza (m2). pH1 = 5 (p1), pH2 = 7 (p2), and pH3 = 8 (p3).

**Table 3:** Interactive effect of AMF and level of pH on some physiological and biochemical characteristics of the cucumber.

Treatments	Phenol of Shoot (mg g^−1^ FW)		DPPH (%)	Proline (µmol g^−1^ FW)	Fe Conc. (mg kg^−1^ DW)	ABA (ng^−1^g FW)	APX (u/mg Pr)	SOD (u/mg Pr)	POX (u/mg Pr)	CAT (u/mg Pr)
m1	p1	87.71 ± 8.86 e	15.10 ± 2.29 abc	2.56 ± 0.04 b	102.68 ± 1.07 a	0.68 ± 0.16 ab	0.46 ± 0.12 a	0.72 ± 0.04 ab	0.43 ± 0.14 a	8.87 ± 0.02 a
	p2	87.88 ± 10.70 d	12.54 ± 2.06 bc	3.51 ± 0.74 a	82.21 ± 2.36 c	0.56 ± 0.13 c	0.39 ± 0.19 b	0.59 ± 0.13 c	0.36 ± 0.21 b	9.36 ± 0.11 a
	p3	71.52 ± 6.10 f	11.10 ± 1.08 c	2.68 ± 0.65 ab	106.20 ± 1.30 a	0.76 ± 0.14 a	0.50 ± 0.13 a	0.817 ± 0.08 a	0.45 ± 0.15 a	8.09 ± 0.06 a
m2	p1	101.05 ± 11.72 b	17.35 ± 2.65 ab	2.97 ± 0.25 ab	95.80 ± 1.96 ab	0.50 ± 0.13 c	0.38 ± 0.09 bc	0.53 ± 0.04 c	0.35 ± 0.11 b	8.80 ± 0.04 a
	p2	135.10 ± 12.14 a	20.09 ± 3.48 a	2.51 ± 0.10 b	83.15 ± 0.98 bc	0.51 ± 0.15 c	0.34 ± 0.04 c	0.55 ± 0.01 c	0.30 ± 0.04 c	10.53 ± 0.09 a
	p3	95.92 ± 9.44 c	19.99 ± 3.47 a	2.79 ± 0.48 ab	78.57 ± 1.28 c	0.59 ± 0.17 bc	0.38 ± 0.08 bc	0.62 ± 0.04 bc	0.35 ± 0.09 b	12.60 ± 0.04 a

Within a column, means followed by the same letter are not significantly different at *p* < 0.05 according to the least significant different test. Treatment includes non-inoculation with mycorrhiza (m1) and inoculation with mycorrhiza (m2). pH1 = 5 (p1), pH2 = 7 (p2), and pH3 = 8 (p3).

**Table 4:** Interactive effect of AMF and Fe concentrations on growth characteristics of the cucumber.

Treatments		Root Volume (mL)	Shoot Fresh Weight (g per Plant)	Root Fresh Weight (g per Plant)	Shoot Diameter (mm)	Shoot Length (cm)	Shoot Dry Weight (g per Plant)	Root Dry Weight (g per Plant)	Root Colonization (%)
	f1	4.17 ± 0.96 a	368.36 ± 25.24 a	134.87 ± 18.98 a	5.35 ± 0.33 a	73.47 ± 2.94 a	32.97 ± 7.77 a	3.67 ± 1.78 a	0 ± 0.00 c
m1	f2	3.40 ± 0.93 a	308.16 ± 26.29 a	98.47 ± 12.69 a	5.25 ± 0.94 a	74.36 ± 4.28 a	33.91 ± 5.18 a	3.56 ± 1.36 a	0 ± 0.00 c
	f3	4.17 ± 0.82 a	345.80 ± 24.29 a	110.60 ± 15.18 a	5.49 ± 0.20 a	74.74 ± 5.08 a	32.32 ± 6.38 a	3.93 ± 1.26 a	0 ± 0.00 c
	f1	4.33 ± 1.71 a	347.36 ± 23.71 a	115.27 ± 18.13 a	5.48 ± 0.48 a	74.84 ± 5.02 a	32.04 ± 7.81 a	3.53 ± 1.82 a	60 ± 4.23 b
m2	f2	4.17 ± 1.42 a	329.16 ± 23.80 a	100.64 ± 10.12 a	5.63 ± 0.26 a	75.12 ± 4.36 a	31.42 ± 9.29 a	3.67 ± 1.02 a	72 ± 4.58 b
	f3	4.17 ± 1.58 a	340.20 ± 28.50 a	114.02 ± 13.35 a	5.37 ± 0.98 a	74.88 ± 5.04 a	30.48 ± 9.89 a	3.32 ± 1.42 a	90 ± 5.20 a

Within a column, means followed by the same letter are not significantly different at *p* < 0.05 according to the least significant different test. Treatment includes non-inoculation with mycorrhiza (m1) and inoculation with mycorrhiza (m2). Fe 0 concentration (f1), Johansson concentration (f2), and amino chelate concentration of Johnson nutrient solution (f3).

**Table 5:** Interactive effect of AMF and Fe concentrations on photosynthetic characteristics of the cucumber.

Treatments		Chlorophyll Index (SPAD Value)	Photosynthesis Rate (μmol(CO_2_) m^2^s^−1^)	Transpiration (mmol(H_2_O)m^2^s^−1^)	Stomata Conductance (mmol(H_2_O) m^2^ s^−1^)	Mesophyll Conductance (µmol)	Photosynthetic Water Use Efficiency (µM mol CO_2_ mol^−1^ H_2_O)
m1	f1	24.35 ± 4.99 bc	5.72 ± 0.26 c	5.88 ± 1.11 d	0.22 ± 0.00 c	0.01 ± 0.007 d	40.59 ± 3.82 b
	f2	20.08 ± 6.09 c	4.62 ± 1.86 d	7.76 ± 1.17 b	0.26 ± 0.01 b	0.01 ± 0.009 e	25.65 ± 2.43 f
	f3	22.81 ± 4.21 bc	4.38 ± 1.09 e	5.44 ± 0.47 e	0.16 ± 0.02 d	0.55 ± 0.001 a	37.30 ± 2.50 d
m2	f1	19.71 ± 7.78 c	4.07 ± 0.77 f	4.63 ± 0.99 f	0.16 ± 0.04 d	0.04 ± 0.004 b	27.43 ± 3.70 e
	f2	30.19 ± 5.16 ab	7.60 ± 1.87 b	7.08 ± 2.53 c	0.22 ± 0.01 c	0.02 ± 0.001 c	52.17 ± 1.85 a
	f3	34.75 ± 7.12 a	7.96 ± 1.17 a	11.39 ± 1.11 a	0.38 ± 0.01 a	0.02 ± 0.007 c	40.46 ± 2.97 c

Within a column, means followed by the same letter are not significantly different at *p* < 0.05 according to the least significant different test. Treatment includes non-inoculation with mycorrhiza (m1) and inoculation with mycorrhiza (m2). Fe 0 concentration (f1), Johansson concentration (f2), and amino chelate concentration of Johnson nutrient solution (f3).

**Table 6:** Interactive effect of AMF and Fe concentrations on some physiological and biochemical characteristics of the cucumber.

Treatments		Phenol of shoot (mg g^−1^ FW)	DPPH (%)	Proline (µmol g^−1^ FW)	Fe conc (mg kg^−1^ DW)	ABA (ng^−1^ g FW)	APX (u/mg Pr)	SOD (u/mg Pr)	POX (u/mg Pr)	CAT (u/mg Pr)
m1	f1	89.42 ± 8.05 c	12.06 ± 2.50 b	1.24 ± 0.20 c	86.14 ± 0.08 c	0.61 ± 0.19 bc	0.39 ± 0.11 b	0.64 ± 0.13 bc	0.36 ± 0.13 b	6.42 ± 0.11 a
	f2	84.45 ± 7.88 d	13.23 ± 2.70 b	4.26 ± 0.23 a	106.03 ± 1.77 a	0.74 ± 0.13 a	0.49 ± 0.14 a	0.79 ± 0.09 a	0.44 ± 0.17 a	8.23 ± 0.06 a
	f3	73.23 ± 10.82 f	13.45 ± 2.85 b	3.27 ± 0.52 b	98.91 ± 0.74 ab	0.65 ± 0.16 ab	0.47 ± 0.13 a	0.69 ± 0.03 ab	0.43 ± 0.16 a	11.68 ± 0.02 a
m2	f1	75.17 ± 12.44 e	20.09 ± 3.16 a	1.24 ± 0.56 c	96.70 ± 0.08 abc	0.52 ± 0.13 d	0.38 ± 0.10 b	0.55 ± 0.04 c	0.35 ± 0.12 b	10.62 ± 0.04 a
	f2	114.12 ± 12.34 b	17.47 ± 2.26 ab	3.61 ± 0.72 ab	89.94 ± 1.08 bc	0.56 ± 0.16 bcd	0.38 ± 0.09 b	0.60 ± 0.03 bc	0.35 ± 0.09 b	9.19 ± 0.04 a
	f3	142.78 ± 12.85 a	19.87 ± 2.46 a	3.43 ± 0.50 ab	70.88 ± 1.22 d	0.52 ± 0.15 cd	0.34 ± 0.05 c	0.56 ± 0.01 c	0.30 ± 0.06 c	12.13 ± 0.05 a

Within a column, means followed by the same letter are not significantly different at *p* < 0.05 according to the least significant different test. Treatment includes non-inoculation with mycorrhiza (m1) and inoculation with mycorrhiza (m2). Fe 0 concentration (f1), Johansson concentration (f2), and amino chelate concentration of Johnson nutrient solution (f3).

**Table 7:** Interactive effect of level of pH and Fe concentrations on characteristics of the cucumber.

Treatments		Root Volume (mL)	Shoot Fresh Weight (g per plant)	Root Fresh Weight (g per Plant)	Shoot Diameter (mm)	Shoot Length (cm)	Shoot Dry Weight (g per Plant)	Root Dry Weight (g per Plant)	Root Colonization (%)
p1	f1	4.56 ± 1.64 a	385.23 ± 29.71 ab	163.80 ± 11.29 a	5.43 ± 0.22 a	73.33 ± 4.29 ab	33.60 ± 7.02 abc	4.08 ± 2.41 a	0 ± 0.00 a
	f2	4.56 ± 1.58 a	400.87 ± 26.59 a	130.90 ± 14.50 ab	5.62 ± 0.24 a	76.13 ± 3.39 ab	39.90 ± 4.66 a	4.15 ± 1.20 a	0 ± 0.00 a
	f3	4.21 ± 1.68 a	416.03 ± 26.30 a	120.87 ± 17.24 ab	5.50 ± 0.21 a	75.90 ± 5.84 ab	33.13 ± 5.98 abc	2.84 ± 1.75 a	0 ± 0.00 a
p2	f1	3.75 ± 0.59 a	331.57 ± 22.77 abcd	97.07 ± 10.55 b	5.27 ± 0.32 a	74.31 ± 4.92 ab	31.26 ± 7.23 bc	2.91 ± 0.74 a	0 ± 0.00 a
	f2	3.40 ± 0.89 a	263.90 ± 22.99 cd	73.73 ± 11.61 b	5.18 ± 1.12 a	75.92 ± 4.44 ab	30.33 ± 4.74 bcd	3.17 ± 1.54 a	0 ± 0.00 a
	f3	4.91 ± 0.29 a	380.10 ± 27.31 ab	131.37 ± 18.09 ab	5.72 ± 0.43 a	76.83 ± 3.99 a	37.98 ± 4.04 ab	4.48 ± 0.55 a	0 ± 0.00 a
p3	f1	4.45 ± 1.64 a	356.77 ± 26.65 abc	114.33 ± 14.22 ab	5.54 ± 0.61 a	74.82 ± 3.44 ab	32.66 ± 6.24 abc	3.80 ± 1.77 a	0 ± 0.00 a
	f2	3.40 ± 0.89 a	291.20 b± 21.16 cd	94.03 ± 11.73 b	5.52 ± 0.44 a	72.16 ± 4.12 ab	27.76 ± 6.65 cd	3.52 ± 0.46 a	0 ± 0.00 a
	f3	3.40 ± 0.89 a	232.87 ± 22.63 d	84.70 ± 13.93 b	5.07 ± 1.06 a	71.72 ± 3.76 b	23.10 ± 5.86 d	3.57 ± 1.05 a	0 ± 0.00 a

Within a column, means followed by the same letter are not significantly different at *p* < 0.05 according to the least significant different test. Treatment includes pH1 = 5 (p1), pH2 = 7 (p2), and pH3 = 8 (p3). Fe 0 concentration (f1), Johansson concentration (f2), and amino chelate concentration of Johnson nutrient solution (f3).

**Table 8:** Interactive effect of level of pH and Fe concentrations on photosynthetic characteristics of the cucumber.

Treatments		Chlorophyll Index (SPAD Value)	Photosynthesis Rate (μmol (CO_2_) m^2^s^−1^)	Transpiration (mmol (H_2_O)m^2^s^−1^)	Stomata Conductance (mmol (H_2_O) m^2^ s^−1^)	Mesophyll Conductance (µmol)	Photosynthetic Water Use Efficiency (µM mol CO_2_ mol^−1^ H_2_O)
p1	f1	23.22 ± 4.84 bcd	5.30 ± 0.36 e	4.24 ± 0.96 g	0.11 ± 0.02 h	0.02 ± 0.001 cd	53.86 ± 3.00 b
	f2	28.61 ± 5.71 abc	6.71 ± 1.98 b	5.41 ± 0.93 f	0.16 ± 0.01 g	0.02 ± 0.002 c	54.36 ± 3.01 a
	f3	32.96 ± 5.71 ab	7.62 ± 2.61 a	9.21 ± 3.62 a	0.310 ± 0.04 a	0.84 ± 0.006 a	42.71 ± 3.89 c
p2	f1	18.68 ± 4.75 cd	4.62 ± 1.54 g	5.38 ± 0.25 f	0.22 ± 0.05 f	0.05 ± 0.04 b	24.31 ± 2.04 h
	f2	28.60 ± 4.31 abc	6.35 ± 1.39 d	9.25 ± 1.14 a	0.28 ± 0.03 b	0.01 ± 0.007 d	36.13 ± 2.01 e
	f3	35.77 ±4.95 a	6.54 ± 1.94 c	8.2617 ± 3.72 b	0.26 ± 0.04 d	0.02 ± 0.003 cd	41.58 ± 2.01 d
p3	f1	24.20 ± 4.88 bcd	4.77 ± 0.85 f	6.15 ± 1.32 e	0.24 ± 0.01 e	0.01 ± 0.004 ef	23.86 ± 1.01 i
	f2	18.19 ± 5.75 d	5.26 ± 1.48 e	7.60 ± 1.33 d	0.27 ± 0.06 c	0.01 ± 0.002 e	26.24 ± 1.01 g
	f3	17.61 ± 3.12 d	4.35 ± 0.68 h	7.78 ± 2.46 c	0.24 ± 0.05 e	0.01 ± 0.008 f	32.34 ± 2.00 f

Within a column, means followed by the same letter are not significantly different at *p* < 0.05 according to the least significant different test. Treatment includes pH1 = 5 (p1), pH2 = 7 (p2), and pH3 = 8 (p3). Fe 0 concentration (f1), Johansson concentration (f2), and amino chelate concentration of Johnson nutrient solution (f3).

**Table 9:** Interactive effect of level of pH and Fe concentrations on some physiological and biochemical characteristics of the cucumber.

Treatments		Phenol of Shoot (mg g^−1^ FW)	DPPH (%)	Proline (µmol g^−1^ FW)	Fe Conc. (mg kg^−1^ DW)	ABA (ng^−1^ g FW)	APX (u/mg Pr)	SOD (u/mg Pr)	POX (u/mg Pr)	CAT (u/mg Pr)
p1	f1	85.34 ± 10.52 f	16.69 ± 3.39 ab	1.24 ± 0.01 c	118.62 ± 0.08 a	0.59 ± 0.12 bc	0.44 ± 0.18 abc	0.63 ± 0.06 b	0.41 ± 0.21 ab	4.57 ± 0.04 a
	f2	112.51 ± 12.60 c	16.56 ± 2.31 ab	3.41 ± 0.55 b	102.20 ± 1.31 b	0.62 ± 0.16 abc	0.43 ± 0.12 abc	0.66 ± 0.05 ab	0.39 ± 0.14 bc	8.51 ± 0.04 a
	f3	85.30 ± 12.63 g	15.42 ± 2.01 ab	3.65 ± 0.82 b	76.89 ± 1.47 cd	0.55 ± 0.21 c	0.39 ± 0.11 c	0.59 ± 0.07 b	0.36 ± 0.13 c	13.43 ± 0.07 a
p2	f1	86.89 ± 12.90 e	19.14 ± 2.76 ab	1.24 ± 0.77 c	67.22 ± 0.08 d	0.42 ± 0.11 d	0.28 ± 0.11 d	0.45 ± 0.06 c	0.26 ± 0.12 d	10.60 ± 0.05 a
	f2	113.75 ± 9.18 b	14.91 ± 2.20 ab	4.80 ± 0.46 a	88.48 ± 1.91 bc	0.60 ± 0.15 bc	0.40 ± 0.12 c	0.64 ± 0.07 b	0.36 ± 0.13 c	7.49 ± 0.06 a
	f3	133.82 ± 11.81 a	14.89 ± 2.15 ab	3.00 ± 0.98 b	92.35 ± 0.28 bc	0.55 ± 0.17 c	0.42 ± 0.12 bc	0.62 ± 0.08 b	0.38 ± 0.13 bc	11.75 ± 0.09 a
p3	f1	74.66 ± 10.20 h	12.39 ± 3.27 b	1.24 ± 0.42 c	88.42 ± 0.08 bc	0.67 ± 0.15 ab	0.44 ± 0.11 ab	0.71 ± 0.03 ab	0.40 ± 0.10 ab	10.38 ± 0.04 a
	f2	71.60 ± 10.65 i	14.58 ± 2.26 ab	3.59 ± 0.79 b	103.28 ± 0.69 ab	0.73 ± 0.12 a	0.47 ± 0.17 a	0.77 ± 0.12 a	0.43 ± 0.10 a	10.13 ± 0.09 a
	f3	104.90 ± 13.16 d	19.66 ± 3.12 a	3.39 ± 0.54 b	85.45 ± 0.89 c	0.62 ± 0.18 abc	0.40 ± 0.13 bc	0.67 ± 0.09 ab	0.36 ± 0.15 c	10.53 ± 0.06 a

Within a column, means followed by the same letter are not significantly different at *p* < 0.05 according to the least significant different test. Treatment includes pH1 = 5 (p1), pH2 = 7 (p2), and pH3 = 8 (p3). Fe 0 concentration (f1), Johansson concentration (f2), and amino chelate concentration of Johnson nutrient solution (f3).

### 2.4. The Spider and Circular Graphs of Interaction of Treatments on the Characteristics of Seedling Cucumber

**Figure 1:**
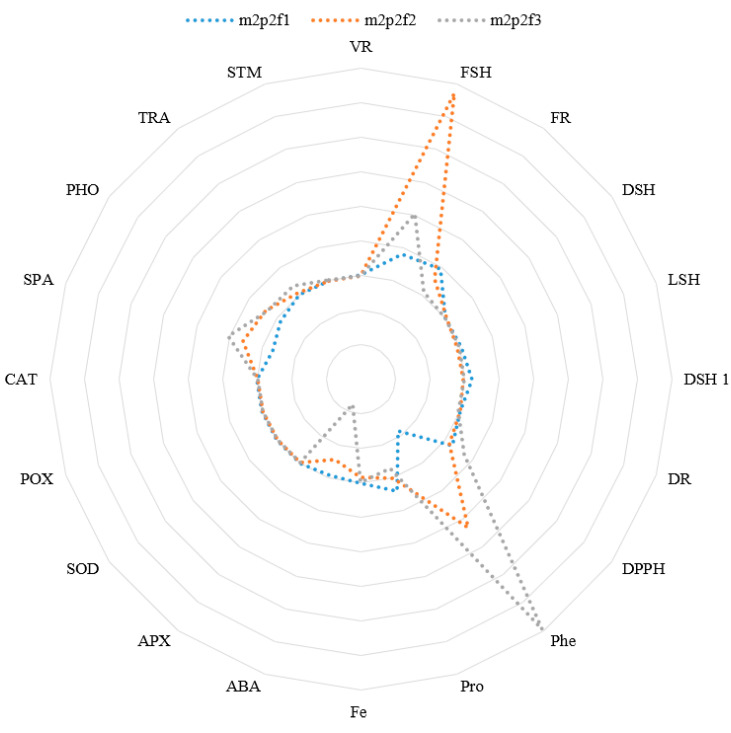
The accumulation of morphology and physiology parameters in cucumber under treatment inoculation with AMF, pH = 7, and different sources of Fe. Root volume (VR), shoot fresh weight (FSH), root fresh weight (FR), shoot diameter (DSH), shoot length (LSH), shoot dry weight (DSH 1), root dry weight (DR), antioxidant capacity (DPPH), phenol of shoot (Phe), proline (Pro), Fe concentration (Fe), abscisic acid (ABA), ascorbate peroxidase (APX), superoxide dismutase (SOD), peroxidase (POX), catalase (CAT), chlorophyll index (SPA), photosynthesis rate (PHO), transpiration (TRA), and stomata conductance (STM). Inoculation mycorrhiza (m2), pH = 7 (p2), Fe 0 concentration (f1), Johansson concentration (f2), and amino chelate concentration of Johnson nutrient solution (f3).

According to spider graphs, the levels of phenol and shoot fresh weight were highest across all treatments compared to other characteristics (Figure 1).

**Figure 2:**
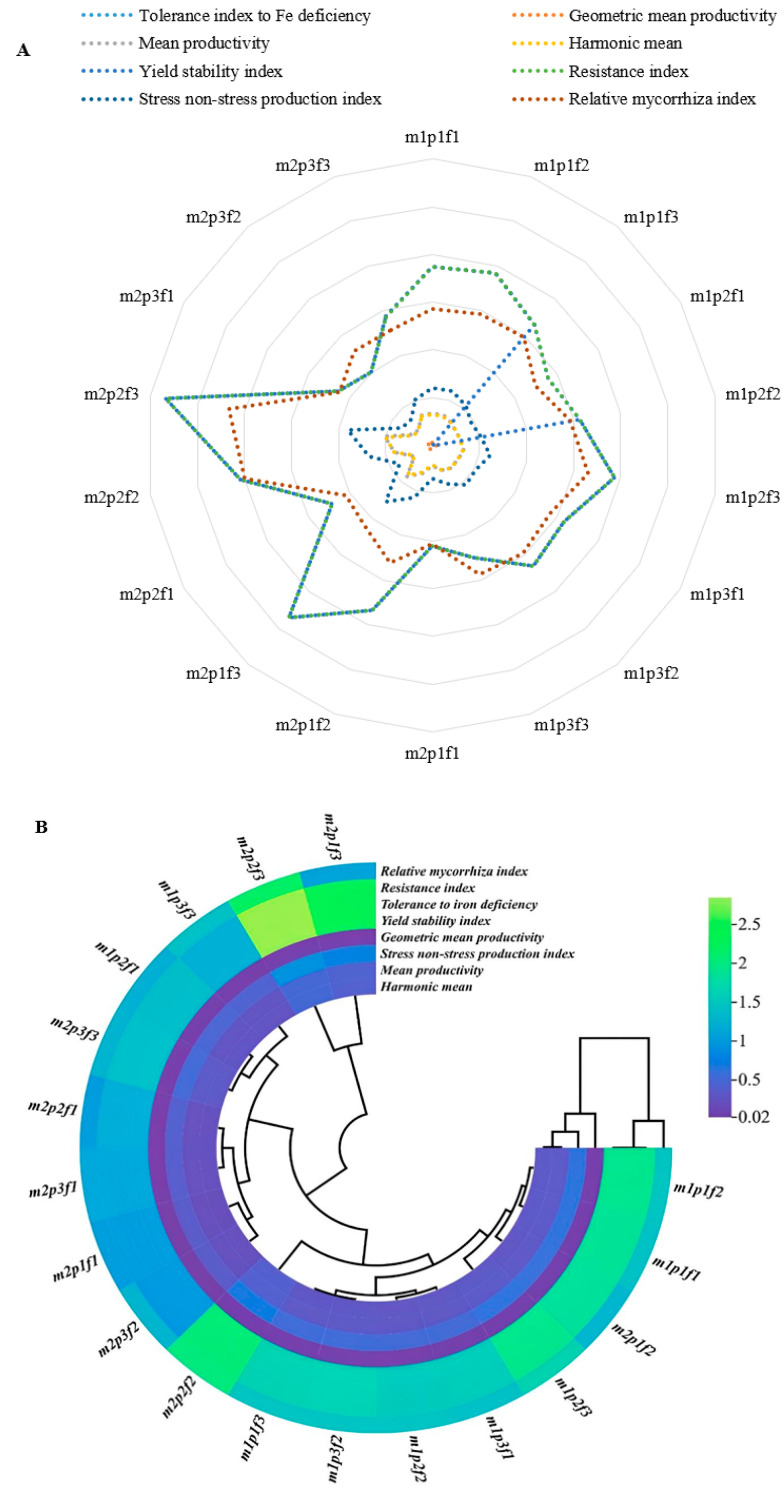
The spider (**A**) and circular plots (**B**) of different tolerance indices to Fe deficiency parameters in cucumber under treatment inoculation AMF, pH, and different sources of Fe. Non-inoculation with AMF (m1) and inoculation with AMF (m2) and iron concentration at three levels: Fe 0 concentration (f1), Johanson concentration (f2), and Johnson’s nutrient solution amino chelate concentration (f3) and pH in three levels: pH1 = 5 (p1), pH2 = 7 (p2) and pH3 = 8 (p3).

Inoculation with AMF led to an increase in most factors, particularly when combined with pH 7 and 2 ppm of the Fe amino chelate, yielding the highest values (Figure 2A). Conversely, the presence of pH 5 and 2 ppm Fe amino chelate also showed significant increases.

The mean productivity genomic trait exhibited a significant negative effect across all treatments (*p* < 0.05). Traits related to the resistance index and tolerance to iron deficiency displayed substantial changes, especially when 2 ppm Fe amino chelate was applied. The most significant impact occurred when 2 ppm of Fe was paired with bacterial inoculation. However, when bacterial inoculation was combined with 2 ppm Fe amino chelate at pH 5 and pH 7, no significant effect was observed, leading to these conditions being grouped together. Overall, resistance traits clustered with the relative mycorrhiza index, while tolerance to iron deficiency and the resistance index were categorized together. This clustering indicates a distinct relationship among these traits, highlighting their interconnected roles in plant responses to iron availability (Figure 2B).

**Figure 3:**
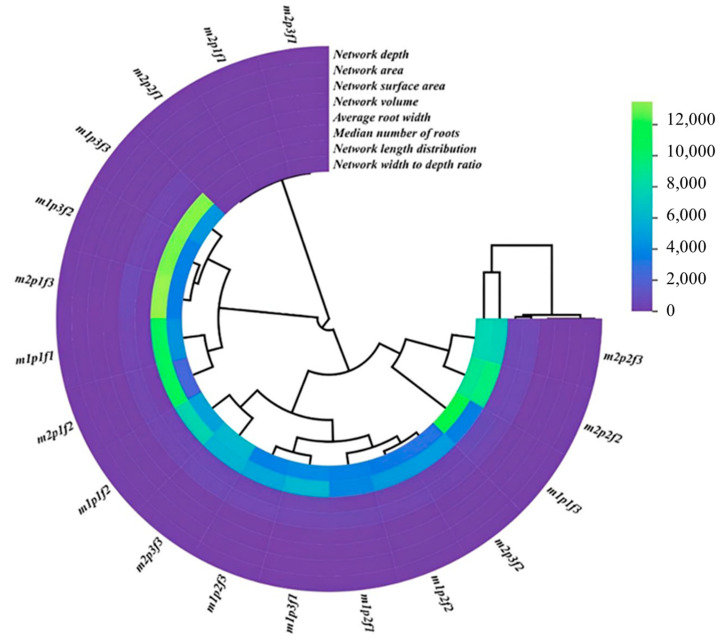
The circular plots of different root network parameters in cucumber under treatment inoculation AMF, pH, and different sources of Fe. Non-inoculation with AMF (m1) and inoculation with AMF (m2) and iron concentration at three levels: Fe 0 concentration (f1), Johanson concentration (f2), and Johnson’s nutrient solution amino chelate concentration (f3), and pH in three levels: pH1 = 5 (p1), pH2 = 7 (p2) and pH3 = 8 (p3).

In contrast, other root traits, such as surface area, volume, and depth, were categorized separately, indicating less responsiveness to different treatments. Among the treatments, the combinations of non-inoculation with AMF at pH 5 with 2 ppm Fe amino chelate, AMF inoculation at pH 7 with 2 ppm Fe amino chelate, non-inoculation with AMF at pH 8 with 2 ppm Fe, and non-inoculation with AMF at pH 8 with 2 ppm Fe amino chelate demonstrated positive changes in both the network width to depth ratio and network length distribution of the roots. This indicates that specific treatments positively affected root structure (Figure 3).

### 2.5. The Principal Component Analysis (PCA) Graph of the Interaction of Treatments on the Characteristics of Seedling Cucumber

**Figure 4:**
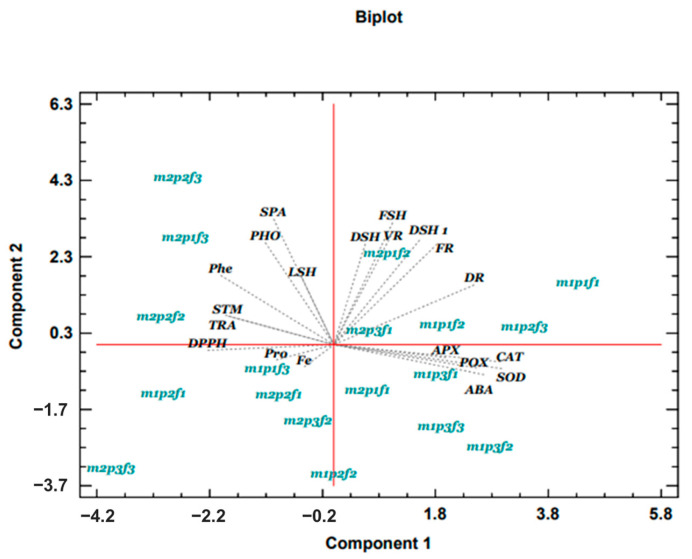
Biplot analysis of the interaction effect of inoculation AMF, pH, and different sources of Fe on the cucumber. Root volume (VR), shoot fresh weight (FSH), root fresh weight (FR), shoot diameter (DSH), shoot length (LSH), shoot dry weight (DSH 1), root dry weight (DR), antioxidant capacity (DPPH), phenol (Phe), proline (Pro), Fe concentration (Fe), abscisic acid (ABA), ascorbate peroxidase (APX), superoxide dismutase (SOD), peroxidase (POX), catalase (CAT), chlorophyll index (SPA), photosynthesis rate (PHO), transpiration (TRA), and stomata conductance (STM).

The PCA diagram indicates that most of the investigated traits increased during bacterial inoculation compared to non-inoculation, with the exception of antioxidant enzymes (CAT, APX, POX, and SOD) and ABA. Specifically, the combination of mycorrhiza inoculation with pH 5 and Fe Johansson concentration significantly enhanced growth traits, including shoot fresh weight, shoot dry weight, root volume, shoot diameter, and root fresh weight (Figure 4).

### 2.6. The Correlation of the Characteristics of Seedling Cucumber

**Figure 5:**
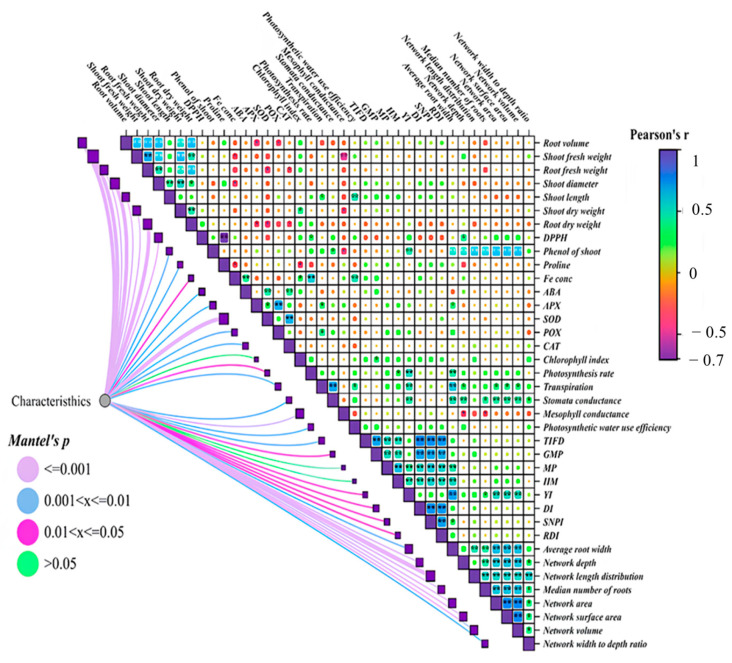
Correlation between the characteristics of cucumber seedlings. * Significant at *p* < 0.05 and ** significant at *p* < 0.01 probability level.

Correlations between various traits reveal interesting relationships in plant physiology. There is a positive correlation between stomatal conductance and transpiration; as stomatal conductance increases, transpiration also rises. Similarly, a positive correlation exists between stem diameter and fresh weight; when the weight increases, the stem diameter also increases. This increase in weight is not solely due to the plant becoming herbaceous; rather, it enhances the diameter and strength of the plant, thereby affecting its fresh weight. Growth traits such as root volume, fresh weight of the stem, fresh weight of the roots, stem diameter, stem length, and dry weight of both stem and roots were strongly correlated with other traits. Root traits, including network area, network surface area, network volume, and width-to-depth ratio, also exhibit high correlations with other characteristics. Overall, many growth and root architecture traits are significantly influenced by other traits, indicating interconnectedness in plant development (Figure 5).

## 3. Discussion

The beneficial effects of amino chelate fertilizers on plant growth can be attributed to the chelating properties of amino acids, which improve the bioavailability of essential micronutrients such as iron. Amino acids in chelated forms protect nutrients against undesirable chemical reactions in the soil and within plant tissues, enhancing their absorption capacity [15,31]. In addition, these fertilizers do not require structural modification for absorption, allowing for quicker recovery in leaves and other tissues under nutrient-deficient conditions [32]. Our study confirmed that the application of amino chelates under pH 5 and neutral conditions significantly increased shoot dry weight and stem length, supporting their efficacy under these specific soil conditions.

In a previous study, Li et al. [33] demonstrated that inoculating plants with *Diversispora versiforme* significantly promoted growth under Fe-deficient conditions, with inoculated plants showing higher biomass production. Wang et al. [34] similarly found that inoculation with *Glomus versiforme* improved plant height, stem diameter, and dry weight of both shoots and roots under Fe deficiency. In our study, inoculation with AMF at pH 5 resulted in increased fresh weights of both shoots and roots, further highlighting the role of AMF in improving nutrient uptake, even in low-Fe environments. The positive effects of AMF on plant growth under nutrient stress are likely due to improved photosynthesis and primary metabolism [35,36]. This aligns with the results of Cartmill et al. [37,38], who showed that cucumber plants inoculated with *Glomus intraradices* had better yield and biomass production under alkaline conditions due to enhanced uptake of minerals such as Fe, P, Mg, Zn, and Mn. Our findings confirmed these findings, as AMF inoculation under pH 7 significantly improved chlorophyll content, photosynthesis rate, and stomatal conductance, contributing to improved plant growth in nutrient-limited environments.

Leaf greenness is a key indicator of plant health, and amino chelates significantly enhance chlorophyll biosynthesis [39,40]. Our findings indicate that other vegetative growth parameters, including plant height and number of leaves, are positively influenced by amino chelates. However, some species exhibit leaf chlorosis following the application of amino chelates. Notably, rice plants treated with Fe and Zn amino chelates showed superior growth compared to those treated with traditional fertilizers, reaffirming the benefits of these compounds [41].

Various components of amino chelates, including nitrogen and micronutrients, contribute to improved chlorophyll biosynthesis and leaf expansion [14,15]. Biostimulation of amino acids in amino chelate formulations plays a crucial role in enhancing leaf morphology and function [15,42].

Arbuscular mycorrhizal fungi colonization has been shown to significantly improve plant water status and stomatal function, particularly under stress conditions like drought, which subsequently increases the efficiency of Photosystem II (PSII). This improvement in PSII efficiency (measured as Fv/Fm) under stress suggests that AMF enhances chloroplast absorption of excitation energy, thereby improving the photochemical capacity of PSII in leaves [43]. Our study found that AMF inoculation at pH 7 resulted in the highest chlorophyll content and photosynthetic efficiency, further supporting the role of AMF in nutrient uptake under optimal conditions. Furthermore, AMF improves the chloroplast cycle and protects pigments from damage caused by stress, such as drought or nutrient deficiency, by enhancing energy absorption and maintaining the structural integrity of the photosystem [44,45]. The accumulation of essential osmolytes and minerals, such as Fe, enhances the chlorophyll content and photosynthetic efficiency of AMF-inoculated plants. In our findings, these beneficial effects were most pronounced at pH 7, which is consistent with the observed increase in photosynthetic efficiency and chlorophyll content and is similar to the results of Abbaspour et al. [46].

Iron is critical for photosynthesis, and iron deficiency can severely affect photosynthetic parameters [47]. Our findings confirmed that non-inoculated plants at pH 8 exhibited lower chlorophyll content and photosynthetic efficiency due to Fe deficiency. Inoculation with AMF mitigated these effects by improving Fe uptake and translocation, emphasizing AMF’s role in enhancing photosynthetic performance in nutrient-limited environments.

In high-alkalinity soils, Fe deficiency is worsened by the reduced availability of Fe in the soil or its uptake by plants [48]. Bicarbonate ions in soil can precipitate Fe, which is less available for plant uptake. The results of the present study confirmed that chlorophyll content and photosynthetic efficiency were lower in non-inoculated plants at pH 8, reflecting the harmful effects of Fe deficiency. However, inoculation with AMF mitigated these effects by enhancing Fe uptake and translocation to the shoot, as observed in a previous study [37]. The increased Fe availability in AMF-treated plants improved chlorophyll synthesis and photosynthetic rates, even under alkaline conditions. The results of the present study emphasize the importance of AMF in alleviating Fe deficiency and improving photosynthetic performance in crops grown under various pH conditions. AMF inoculation not only enhances chlorophyll biosynthesis but also boosts the overall efficiency of the photosynthetic apparatus by improving nutrient uptake and maintaining water status. These findings align with previous studies and suggest that integrating AMF with traditional fertilization strategies can significantly improve plant resilience to environmental stress, particularly in alkaline soils where Fe availability is limited.

Fouad et al. [49] demonstrated that AMF-inoculated plants under moderate stress exhibit higher activities of key antioxidant enzymes such as SOD, APX, and guaiacol peroxidase (GPX). These enzymes are crucial in mitigating oxidative stress by neutralizing reactive oxygen species (ROS) generated during environmental stress, such as drought. The role of AMF in enhancing these enzyme activities is attributed to its ability to improve nutrient uptake (such as Zn and Cu), which are critical cofactors for these antioxidant enzymes [50]. AMF’s contribution to ion transfer and gene activation has been suggested as a mechanism by which the expression of these enzymes is increased under stress [51]. In contrast to previous studies, our findings showed the highest levels of SOD, APX, and GPX activity under non-AMF-inoculated conditions at pH 8. This suggests that, in this case, the absence of AMF and the stress induced by alkaline pH might have triggered an increased production of ROS, resulting in stronger activation of antioxidant defense mechanisms in non-inoculated plants. This divergence from established studies could be due to specific pH conditions, which might have overshadowed the protective role of AMF and led to a more robust intrinsic response from non-inoculated plants.

Amino chelates, particularly Fe-chelated compounds, have also been shown to mitigate oxidative stress by enhancing antioxidant enzyme activity. For example, Fe-amino acid chelates, such as Fe-Gly2, increase CAT and APX activities in plants under salt stress compared with traditional Fe-EDTA treatment [52,53]. In your study, the application of Fe-amino chelates under non-AMF-inoculated conditions resulted in increased activity of CAT, APX, and SOD, further supporting the potential of amino chelates to enhance plant defenses against oxidative stress.

Cartmill et al. [38] highlighted the role of AMF in enhancing plant tolerance to alkaline conditions through various mechanisms. In particular, the tolerance of vinca plants inoculated with a mix of *Glomus* species was linked to increased phosphorus (P) uptake, supported by higher soluble phosphatase activity under moderate bicarbonate (HCO_3_^−^) concentrations. Furthermore, these inoculated plants maintained nutrient balance in the leaves, even as bicarbonate levels increased up to 10 mM, and exhibited enhanced antioxidant activity. This improved antioxidant defense is likely due to the improved micronutrient status provided by AMF inoculation, which helps detoxify ROS under stressful conditions [38]. The findings emphasize the multi-faceted role of AMF, not only in nutrient uptake (like P) but also in improving the plant’s oxidative stress response by ensuring a more balanced micronutrient profile, particularly under adverse environmental conditions such as alkalinity. This finding aligns with a broader understanding of AMF’s contribution to plant resilience in nutrient-deficient or stress-prone environments.

AMF-colonized plants exhibited lower total root length and specific root length but greater mean root diameter and root tissue density than non-inoculated plants under iron and phosphorus conditions. This suggests that although AMF reduces total root length, it enhances root functionality and complexity. For instance, our findings align with the observation that AMF colonization can positively influence root architecture, which is crucial for nutrient uptake. Although Pang et al. [54] noted variability in AMF’s impact on root architecture based on environmental conditions and nutrient availability, our results emphasize the specific benefits observed under the conditions of our study.

Hooker et al. [55] also found that AMF colonization did not affect total root length but significantly increased the lengths of individual secondary and tertiary roots. This result supports our observation of reduced overall root length and highlights the enhanced complexity of AMF-colonized plants. Additionally, Chen et al. [56] reported significant increases in root morphological parameters in mycorrhizal seedlings under low P conditions, indicating that AMF can improve root traits depending on nutrient availability.

Furthermore, Ramírez-Flores et al. [57] demonstrated that AMF colonization is associated with increased root system size and branching, which enhance root solidity. These findings support our hypothesis that AMF not only enhances nutrient uptake capacity but positively influences root system architecture, which explains the increased root tissue density and mean root diameter observed in our study.

It is well established that AMF stimulates the synthesis of phenolic compounds in host plants, which is particularly critical when plants are exposed to abiotic or biotic stress [58]. The phenomenon known as mycorrhizal-induced resistance (MIR) describes the protective effects conferred by AMF on plants. This resistance is a result of metabolic and genetic rearrangements triggered by AMF colonization and affects both primary and secondary plant metabolism [59]. One key aspect of this metabolic alteration is the enhanced synthesis of defense enzymes, which play vital roles in the physiological and biochemical resistance of plants. Studies by Chen et al. [60] have shown that AMF effectively induces the accumulation of phenol and flavonoid compounds known for their potent antioxidant properties. These phenolic compounds act as free radical scavengers and reducing agents that protect plants from oxidative stress. This is consistent with our findings, in which AMF inoculation at pH 2 with iron amino chelate led to a significant increase in phenol content in plant stems compared with the other treatments. The elevated phenol levels observed in AMF-colonized plants suggest that AMF enhances the plant’s defense system under stressful conditions, likely contributing to greater stress resilience and overall plant health. The increased phenolic content in AMF-treated plants, particularly under low pH and iron amino chelate treatments, underscores the important role of AMF in enhancing plant stress tolerance. These findings are consistent with the concept of mycorrhizal-induced resistance, in which AMF not only improves nutrient uptake but also fortifies the plant’s biochemical defenses, making it more resilient to environmental challenges.

Rosielle and Hamblin [61] defined the stress tolerance index (STI) and mean productivity (MP) as metrics for assessing yield differences between stress and non-stress conditions. İlker et al. [62] emphasized that parameters like MP, geometric mean productivity (GMP), and STI are effective for selecting high-yielding wheat genotypes under varying conditions. Furthermore, indices such as tolerance (TOL) and stress susceptibility index (SSI) are more suitable for evaluating tolerance levels, and correlations between grain yield and drought tolerance indices can effectively identify optimal cultivars [63]. Malekshahi et al. [64] also reported significant positive correlations among GMP, MP, and STI with stress yield, reinforcing the idea that ideal indices should correlate well with yield under both stress and non-stress conditions. However, Ehdaie and Shakiba [65] found no correlation between stress susceptibility and yield in wheat under optimal conditions, highlighting the complexity of these relationships.

In our study, AMF inoculation significantly increased all indices related to iron deficiency resistance, including the Fe deficiency index (TIFD), GMP, mean productivity (MP), harmonic mean (HM), yield stability index (YSI), resistance index (RI), stress non-stress production index (SNPI), and relative mycorrhizal index (RMI), compared with non-inoculated conditions. These results suggest that AMF inoculation enhances plant resistance to nutrient deficiencies, particularly iron and phosphorus, by improving nutrient absorption, ultimately leading to higher yield production.

## 4. Materials and Methods

### 4.1. Experimental Design

The experiment was conducted in a greenhouse at the Department of Horticulture at Isfahan University of Technology. The study was conducted as a factorial experiment based on a complete randomized design (CRD) with 3 replications. Treatments included mycorrhiza (*Glomus mossea*) inoculation, different Fe concentrations, and different pH levels. Mycorrhiza at two levels: non-inoculation with AMF (m1) and inoculation with AMF (m2), and iron concentration at three levels: Fe 0 concentration (f1), Johanson concentration (f2), and Johnson’s nutrient solution amino chelate concentration (f3). For f2, the iron concentration based on the Johanson stock solution was 2 ppm. Similarly, for f3, the concentration was also 2 ppm, but in the form of amino chelate and pH in three levels: pH1 = 5 (p1), pH2 = 7 (p2), and pH3 = 8 (p3) [66]. The treatment abbreviations used are presented in Table 10.

### 4.2. Plant Growing Conditions

At the beginning of the experiment, the planting bed (sand) was sterilized at 80 °C for 24 h. Mycorrhiza fungi (*Glomus mossea*), which have a population of 100 spores per plant [67], were obtained from the Soil and Water Research Center of Iran. Then, an inoculum containing 100 spores per plant was added to the soil, which should have been treated at a rate of 5% by weight and mixed well. To simulate equal microflora in non-inoculation and AMF treatments, filtrate of the mycorrhizal inoculum (without any spores) was added to non-inoculation treatments. Then, cucumber seeds (*Cucumis sativus* var. Super N3) plants were grown in this environment. After plant establishment, pH and iron treatments were used. To reduce the acidity of the nutrient solution, 1 mM hydrochloric acid and 4 normal potassium hydroxide were added to increase the acidity of the food solution. The pots were irrigated every other day with 500 mL of Johnson’s solution, according to the treatment instructions.

Johnson nutrient solution including (mM): MgSO_4_: 2, KH_2_PO_4_:1, H_3_BO_3_:50, MnCl_2_: 10, CaCl_2_: 1, MnSO_4_: 10, CuSO_4_: 1.5, ZnSO_4_: 0.8, Na_2_MoO_4_: 0.4, Co (NO_3_)_2_: 0.1, KNO_3_: 10, and 2 ppm Fe is in the form of FeCl_3_ and EDTA [68].

### 4.3. Preparation of Chemicals

An iron amino chelate was prepared based on a Johnson solution using the amino acid tryptophan as a complexing agent. Two mmol of amino acid tryptophan were dissolved in 5 mL of distilled water, and then the nutrient solution was slowly added to 1% iron amino chelate solution along with 2 mL of distilled water. The mixture was heated for 2 h while stirring vigorously. Evaporation of the solvent at room temperature resulted in the production of iron amino chelates. The produced product was washed with ethanol and air-dried [52].

### 4.4. Measured Parameters

#### 4.4.1. Growth Trait Assay

At the end of the experiment, and before the appearance of the first flower, when the plants were still in the vegetative stage, we harvested and washed the entire plants from each treatment group, ensuring three replicates for the measurement of morphological and growth traits. Shoots were separated from roots using a steel blade and dried for two days in a conventional oven at 70 °C to achieve a constant weight. The fresh weight (FW) and dry weight (DW) of shoots and roots were calculated, as well as their ratios. The lengths of the roots and shoots were determined using a ruler, and the stem diameters were measured using a digital caliper (Model 16 ER 0-6; Mahr GmbH, Göttingen, Germany). A change in water volume was used to calculate the root volume [69]. Roots were then carefully arranged for image capture using a scanner, followed by an analysis of root system architecture using the WinRHIZO1 image analysis system (V4.1c, Re’gent Instruments, Quebec, QC, Canada) [70]. Using this system, the total root length, root surface area, root volume, and average root diameters of different sizes were automatically analyzed.

#### 4.4.2. AMF Colonization

To determine AMF colonization in roots, we first washed root systems with a free soil–sand mixture before clearing them with KOH (10% *w*/*v*) at 95 °C for 1 h. Next, we acidified roots with HCl (1% *w*/*v*) for 1 h, then stained them with 0.05% *w*/*v* trypan blue in lactoglycerol (8:1:1 lactic acid, glycerol, and water) for 20 min Phillips and Hayman [71]. Finally, we quantified the percentage of mycorrhizal root colonization using the guideline intersection method of Giovannetti and Mosse [72].

#### 4.4.3. Chlorophyll Content and Transpiration Assay

Before entering the flowering stage, chlorophyll content and transpiration were measured in mature middle leaves using a chlorophyll meter (SPAD-502 plus, Minolta, Japan). Three readings were made from different leaves of each plant for the sum of nine measurements for each duplicate [73]. At the same time, photosynthetic parameters (photosynthesis rate and transpiration) were measured using fully expanded leaves by an infrared-calibrated portable gas exchange system (LCi, ADC Bioscientific Ltd., Hoddesdon, UK) over a 2-h period of light saturation intensity from 11 am to 1 pm.

#### 4.4.4. Phenol Content

The total phenolic content of the samples was evaluated using the method developed by Folin-Ciocalteu [74]. Folin-Ciocalteu reagent was diluted with distilled water (10 times). The soluble cucumber extract (20 mL) was blended with the diluted Folin-Ciocalteu reagent (1 mL), sodium bicarbonate solution (7.5%; 1 mL), and distilled water (1 mL). The resulting mixture was maintained at room temperature for 15 min. The absorbance was recorded at 730 nm using a spectrophotometer (V-530, JASCO, Tokyo, Japan). The calibration curves of the samples were equivalent to gallic acid equivalents (GAE). The total phenolic content was reported as gallic acid equivalents (mg) of 100 g of fresh cucumber.

#### 4.4.5. Antioxidant Activity

The antioxidant activity of cucumber leaves was determined by Koleva et al. [75]. One g of sample was dissolved in 5 mL of methanol stock, and 1.4 mL of this solution was blended with 0.6 mL of DPPH solution. After 30 min, the absorbance of the solution was recorded at 515 nm using a spectrophotometer (UV 160A- Shimadzu Corp., Kyoto, Japan) against methanol as a blank. The 0.2 mM DPPH solution in methanol was used as a stock DPPH to determine the free radical-scavenging activity of the samples.

#### 4.4.6. Proline Concentration

The ninhydrin test was used to determine the concentration of proline. Sulfosalicylic acid (3%) was used to homogenize leaf samples at 4 °C. The resulting solution was incubated and centrifuged (5000 rpm for 20 min). The supernatant was blended with ninhydrin (2.5%), phosphoric acid (60%; *v*/*v*), and glacial acetic acid (100%; 1 mL). The absorbance was recorded at 518 nm [76].

#### 4.4.7. Iron Concentration

The leaf tissues were fragmented into pieces and subjected to oven drying at 60 °C for 48 h. The leaf samples were pulverized using a grinding machine, placed into small plastic bags, and stored in a dry environment. During the analysis, 1 g of each sample was transferred to a crucible and then subjected to a temperature of 550 °C in a furnace for 6 h to obtain the dry ash. The complete digestion of the ashes was performed by adding drops of 2 N HNO_3_ and, subsequently, 1 N HCl solution. The mixture was subjected to heating at 70 °C for 20 min until a solution of a pale hue was achieved. The remaining substance was cleaned and strained into a 50 mL container using purified water and Whatman 4 filter paper. The concentration of Fe in plant tissues was measured using atomic absorption spectroscopy (PerkinElmer AAnalyst 700; Waltham, MA, USA) [77].

#### 4.4.8. ABA Content of Leaves

One gram of the fresh leaves was added to 10 mL of 80% methanol, and 0.1 g of polyvinylpyrrolidone was homogenized at 4 °C. The sample was then centrifuged at 4000× *g* for 15 min. Once the pH reached 8, the supernatant was removed. After evaporating the methanol, 5 mL of deionized water was added and dissolved twice. Ethyl acetate was then added and re-vaporized. The remaining residue was then dissolved by adding 3 mL of 0.1 M acetic acid and 1 mL of an 80% methanol solution. Then, an HPLC system’s reverse-phase column (Diamonsic, C18, 5 m), measuring 25 cm in length and 4.6 mm in diameter, was filled with ABA using a 0.45-mm filter (Unicam-Crystal-200, Cambridge, UK). In addition to using a diode-array detector, a gradient phase of methanol-acetic acid (3–97%) was applied at a rate of 4 mL min^−1^. The output peak was then calibrated, and the sample extraction level was determined using an ABA standard with a purity of 99.97% (Sigma factory builds, Darmstadt, Germany). The extracted sample value was specified according to the area under the curve and how it behaved in the output peaks [78].

#### 4.4.9. Antioxidant Enzymes Assay

Sample (1 g) was homogenized in 5 mL of phosphate-buffered saline (PBS; 50 mM; pH 7.8 for SOD and pH 7 for CAT, APX, POD), centrifuged at 12,000× *g* for 20 min at 4 °C, and the supernatant was used for enzymatic measurements [79]. The inhibition of nitroblue tetrazolium (NBT) reduction was used for SOD measurements. The reduction of NBT to blue formazan by superoxide radicals occurred at 560 nm. The reaction mixture comprised 1.9 mL of phosphate buffer [pH 7.8], methionine, NBT, and riboflavin, with suitably diluted erythrocyte hemolysate in a total volume of 3 mL. The total reaction mixture for CAT measurements included sodium phosphate buffer and 10 mM H_2_O_2_ and was initiated by adding 100 µL enzyme extract and reading at 240 nm for 30 s. To estimate POD activity, 25 mM PBS (pH 7.0), 0.05% guaiacol, 1.0 mM H_2_O_2_, and 0.1 mL of the sample extract were mixed, and guaiacol oxidation was recorded at 470 nm for 3 min. To measure ascorbate peroxidase activity, 50 mM PBS (pH 7.0), 0.5 mM AsA (vitamin C), 0.1 mM H_2_O_2_, and 200 µL of sample extract were added together, and the resultant extract was observed spectrophotometrically at 290 nm wavelength.

#### 4.4.10. Calculation of Indices

Eight tolerance indices to Fe deficiency were calculated using the following relationships:

1. TIFD = ($Y_{M}\times Y_{C})/({{\bar{Y}}_{C})}^{2}$, high TIFD value indicates tolerance to iron deficiency;
2. GMP = $\sqrt{(Y_{M})(}Y_{C})$, high value of this index will be more desirable;
3. MP = $(Y_{M}+Y_{C})/2$, high value of this index will be more desirable;
4. HM = $\left[2(Y_{C})(Y_{M})]/(Y_{C}+Y_{M}\right)$, high HM value will be more desirable;
5. YSI = $Y_{M}/Y_{C}$, high values of YSI can be considered stable under Fe deficiency and without Fe deficiency;
6. RI = ${[Y}_{M}\times$ ${(Y}_{M}/Y_{C})]/{\bar{Y}}_{M}$;
7. SNPI = [${(Y}_{C}+Y_{M})/{\left(Y_{C}-Y_{M}\right)}^{1/3}\times {\left(Y_{C}\times Y_{M}\times Y_{M}\right)}^{1/3}$;
8. RMI = [($Y_{M}/Y_{C})/(\;{\bar{Y}}_{M}/{\bar{Y}}_{C})]$.

where Y_M_ and Y_C_ represent yield in mycorrhizal and non-mycorrhizal conditions, respectively. Also, ${\bar{Y}}_{M}$ and ${\bar{Y}}_{C}\;$ are mean yield of cucumber in mycorrhizal and non-mycorrhizal conditions, respectively [63].

### 4.5. Statistical Analysis

Data were analyzed using Statistix 8 (Tallahassee, FL, USA). All data were subjected to a two-way analysis of variance, and the means were compared for significance using the least significant difference (LSD) test at *p* < 0.05. Spider graphs were created using Excel 2013. PCA was performed using Statgraphics Centurion Version XVI. Circular diagrams and correlations between traits were created using the website https://www.chiplot.online/.

## 5. Conclusions

This study highlights the substantial benefits of mycorrhizal inoculation and iron amino chelators in enhancing cucumber growth, particularly under neutral pH conditions (pH 7). The combination of *Glomus mosseae* inoculation and iron in the form of amino chelates significantly improved key physiological traits, including chlorophyll content, photosynthesis rate, stomatal conductance, and phenolic compound accumulation. The synergy between mycorrhiza and iron amino chelates notably increased the activity of antioxidant enzymes such as SOD, POD, CAT, and APX, which mitigated oxidative stress and promoted plant health. The results indicate that mycorrhizal inoculation enhances the resistance of plants to iron deficiency, effectively reducing the demand for elevated antioxidant activity by improving nutrient uptake and minimizing stress. Among the tested treatments, mycorrhiza combined with 2 ppm of a Fe amino chelate at pH 7 was the most effective in improving not only photosynthesis and antioxidant defense but also shoot and root biomass. Additionally, the study revealed significant enhancements in root and stem fresh weights at pH 5, suggesting that lower pH levels facilitate better nutrient solubility and uptake, promoting root development and overall plant health. Furthermore, the increase in ABA levels and antioxidant enzyme activities observed in the absence of mycorrhiza at pH 7 underscores the protective role of AMF under neutral pH conditions. These findings emphasize the potential of integrating mycorrhizal inoculation with iron amino chelate-based fertilizers to improve crop productivity and stress tolerance, particularly in nutrient-limited environments. In conclusion, this research offers a sustainable solution for optimizing nutrient use efficiency and enhancing the performance of greenhouse crops, such as cucumbers, under challenging soil conditions. By considering multiple physiological parameters, we provide a comprehensive understanding of how mycorrhizal inoculation and iron supplementation can synergistically improve plant resilience and contribute to sustainable agricultural practices that address the global challenges of food security and soil health.

## Figures and Tables

**Table 10:** Abbreviation of treatments.

Treatment Factor	Levels	Codes
Mycorrhiza inoculation (AMF)	Non-inoculation (Control)	m1
	Inoculation with AMF	m2
Iron concentration	Fe 0 (Control)	f1
	Johnson’s concentration (2 ppm Fe)	f2
	Johnson’s nutrient solution amino chelate (2 ppm Fe)	f3
pH levels	pH 5	p1
	pH 7	p2
	pH 8	p3

## Data Availability

The data presented in this study are available on request from the corresponding authors.

## Supplementary Materials

The following supporting information can be downloaded at www.mdpi.com/article/10.3390/plants14030341/s1, Table S1. Analysis of variance of AMF, level pH and concentration Fe on growth characteristics and some physiological characteristic of cucumber. Table S2. Analysis of variance of AMF, level pH and concentration Fe on biochemical characteristics and photosynthetic index of cucumber. Table S3. Analysis of variance of AMF, level pH and concentration Fe on stress index of cucumber. Table S4. Analysis of variance of AMF, level pH and concentration Fe on Network root characteristics of cucumber. Table S5. The main effect of experimental treatment (AMF, pH and Fe) on growth characteristics and some physiological characteristic of cucumber. Table S6. The main effect of experimental treatment (AMF, pH and Fe) on biochemical characteristics and photosynthetic index of cucumber. Table S7. The main effect of experimental treatment (AMF, pH and Fe) on stress index of cucumber. Table S8. The main effect of experimental treatment (AMF, pH and Fe) on Network root characteristics of cucumber.
