# Supplementary material for: Impact of Mycorrhiza Inoculations and Iron Amino Chelate on Growth and Physiological Changes of Cucumber Seedlings Across Different pH Levels
Source: Plants (Basel). 2025 Jan 23;14(3):341. doi: 10.3390/plants14030341 (PMC11819710; doi:10.3390/plants14030341)
Supplement: Supplementary file 1 [file plants-14-00341-s001.zip › plants-3343216-supplementary.pdf]

Table S1. Analysis of variance of AMF, level pH and concentration Fe on growth characteristics and some physiological characteristic of cucumber

Source of variation	df	Root volume	Shoot fresh weight	Root fresh weight	Shoot diameter	Shoot length	Shoot dry weight	Root dry weight	Phenol of shoot	DPPH	Proline
AMF	1	1.30667 n.s	47 n.s	294 n.s	0.24536 n.s	7.7369 n.s	41.466 n.s	0.61014 n.s	10830.1 *	523.54 **	47.1895 n.s
pH	2	2.2413 n.s	54513.5 **	9316.21 *	0.1009 n.s	38.9673 n.s	280.325 **	0.13502 n.s	3531.87 *	3.2 n.s	11.0246 n.s
Fe	2	1.12519 n.s	7049.9 n.s	2928.68 n.s	0.00285 n.s	2.3502 n.s	8.495 n.s	0.00436 n.s	3076.36 *	7.72 n.s	65.077 n.s
AMF × pH	2	0.57167 n.s	19176.7 *	2716.02 *	0.71235 n.s	46.5634 **	37.677 *	0.30199 n.s	1342.5 *	55.61 *	25.2827 n.s
AMF × Fe	2	0.76222 n.s	2031.5 n.s	754.38 n.s	0.27184 n.s	1.699 n.s	2.745 n.s	0.59381 n.s	7906.43 *	16.23 *	18.6248 n.s
pH × Fe	4	2.55435 n.s	18931.4 **	3258.77 *	0.48716 n.s	19.4027 *	159.556 *	3.79478 n.s	1880.51 *	57.34 *	41.2383 n.s
AMF × pH × Fe	4	1.21139 *	13600.4 *	4345.37 *	0.3763 *	2.0262 *	54.653 *	0.94879 n.s	1186.38 *	51.36 *	38.4303 n.s
Error	34	1.71126	8070.9	2646.99	0.38122	17.9031	43.748	2.4361	0.00112	34.13	59.5423
CV		32.11	26.44	45.81	11.37	5.67	20.54	43.16	0.03	36.44	79.44

ns: not significant, * significant at p<0.05 and ** significant at p<0.01 probability level, df: degree of freedom

Table S2. Analysis of variance of AMF, level pH and concentration Fe on biochemical characteristics and photosynthetic index of cucumber

Source of variation	df	Fe conc.	ABA	APX	SOD	POX	CAT	Chlorophyll index	Photosynthesis rate	Transpiration	Stomata conductance	Mesophyll conductance	Photosynthetic water use efficiency
AMF	1	0.3572 n.s	1689.34 **	0.23968 *	0.09731 *	0.2532 **	0.08943 *	454.952 *	35.966 *	24.3748 *	0.02323 *	0.38002 *	409.476 *
pH	2	0.414 n.s	1245.36 **	0.08922 **	0.02538 *	0.0979 **	0.02297 *	382.751 *	13.9628 *	8.3974 *	0.01986 *	0.44424 *	2489.3 *
Fe	2	36.1558 n.s	770.74 *	0.03843 *	0.00992 **	0.04425 *	0.00725 *	205.374 **	9.347 *	47.0003 *	0.02832 *	0.42722 *	143.266 *
AMF × pH	2	2.4729 **	980.73 **	0.02122 *	0.00653 *	0.03368 **	0.00172 *	70.374 *	42.8156 *	11.8194 *	0.02841 *	0.45597 *	795.971 *
AMF × Fe	2	0.8274 *	1757.13 **	0.0082 *	0.01854 **	0.01152 *	0.01509 *	371.89 *	36.8362 *	72.2139 *	0.10801 *	0.45389 *	1789.4 *
pH × Fe	4	1.8533 **	1763.87 **	0.02188 **	0.01642 **	0.02389 *	0.01514 *	229.729 *	3.4606 *	11.2852 *	0.02141 *	0.45872 *	349.889 *
AMF × pH × Fe	4	1.8621 **	1316.37 **	0.03504 **	0.00917 **	0.04401 *	0.00697 *	82.475 *	15.199 *	1.2986 *	0.01231 *	0.43657 *	370.44 *
Error	34	0.8456	175.11	0.00882	0.00142	0.01193	0.00051	78.5	0.0011	0.0011	0.00001	0.00001	0.00115
CV		32.34	14.47	15.52	9.1	16.98	6	34.99	0.58	0.48	1.17	2.27	0.09

ns: not significant, * significant at p<0.05 and ** significant at p<0.01 probability level, df: degree of freedom

Table S3. Analysis of variance of AMF, level pH and concentration Fe on stress index of cucumber

Source of variation	df	Tolerance index to Fe deficiency (TIFD)	Geometric mean productivity (GMP)	Mean productivity (MP)	Harmonic mean (HM)	Yield stability index (YSI)	Resistance index (RI)	Stress non-stress production index (SNPI)	Relative mycorrhiza index (RMI)
AMF	1	0.00156 n.s	0.00001 n.s	0.00107 n.s	0.00042 n.s	0.00156 n.s	0.00156 n.s	0.00098 n.s	0.01245 n.s
pH	2	1.30162 **	0.00056 **	0.01958 *	0.01784 *	1.30162 **	1.30162 **	0.10504 **	0.30944 **
Fe	2	1.2752 **	0.0004 **	0.04451 *	0.03922 *	1.2752 **	1.2752 **	0.1406 *	1.02127 **
AMF × pH	2	0.58851 *	0.00032 *	0.01457 *	0.01457 *	0.58851 *	0.58851 *	0.05162 **	0.27898 **
AMF × Fe	2	1.33867 **	0.00041 **	0.04891 *	0.04407 *	1.33867 **	1.33867 **	0.14654 *	0.45201 *
pH × Fe	4	0.47892 *	0.00025 *	0.00645 **	0.00627 **	0.47892 *	0.47892 *	0.03448 *	0.23702 **
AMF × pH × Fe	4	0.12927 *	0.00007 *	0.0028 **	0.00314 *	0.12927 *	0.12927 *	0.01071 *	0.12645 *
Error	34	0.13251	0.00007	0.00115	0.00095	0.13251	0.13251	0.00973	0.10232
CV		22.03	24.41	10.74	10.05	22.03	22.03	18.25	21.79

ns: not significant, * significant at $p<0.05$ and ** significant at $p<0.01$ probability level, df: degree of freedom

Table S4. Analysis of variance of AMF, level pH and concentration Fe on Network root characteristics of cucumber

Source of variation	df	Average Root Width (Diameter)	Network Depth	Network Length Distribution	Median Number of Roots	Network Area	Network Surface Area	Network Volume	Network Width to Depth Ratio
AMF	1	38915 *	0.25079 n.s	4.24E+07 n.s	129911 *	1.64501 *	1.68328 *	1.69212 *	2.55E+07 n.s
pH	2	21359 **	0.12275 n.s	3.17E+07 n.s	4488 n.s	0.00082 n.s	0.00121 n.s	0.0012 n.s	5723326 n.s
Fe	2	383405 *	0.07776 n.s	1.43E+08 n.s	282597 *	1.641 *	1.68452 *	1.6923 *	8.01E+07 **
AMF × pH	2	8220 *	0.00327 *	9.78E+07 *	201314 **	0.00218 *	0.00138 *	0.0013 *	5.84E+07 **
AMF × Fe	2	441451 *	0.24004 **	8.80E+07 *	81046 *	1.68028 *	1.69761 *	1.69797 *	2.67E+07 *
pH × Fe	4	3795 **	0.09923 *	4228576 n.s	28241 *	0.00167 *	0.00133 *	0.0013 *	8038600 *
AMF × pH × Fe	4	9249 **	0.38226 *	5.14E+07 *	55546 *	0.00106 *	0.00118 *	0.00124 *	1.75E+07 *
Error	34	1756	0.09733	5.37E+07	20072	0.01991	0.01994	0.01995	8607242
CV		11.52	28.94	1.11E+02	54.27	74.61	78.14	78.74	6.92E+01

ns: not significant, * significant at $p<0.05$ and ** significant at $p<0.01$ probability level, df: degree of freedom

Table S5. The main effect of experimental treatment (AMF, pH and Fe) on growth characteristics and some physiological characteristic of cucumber

Treatment	Root volume (ml)	Shoot fresh weight (g per plant)	Root fresh weight (g per plant)	Shoot diameter (mm)	Shoot length (cm)	Shoot dry weight (g per plant)	Root dry weight (g per plant)	Phenol of shoot (mg g^{-1} FW)	DPPH (%)	Proline (µmolg^{-1} FW)
AMF1	3.9185 a	340.77 a	114.64 a	5.3650 a	74.194 a	33.071 a	3.7230 a	82.37 b	12.919 b	8.779 a
AMF2	4.2296 a	338.90 a	109.98 a	5.4998 a	74.951 a	31.319 a	3.5104 a	110.69 a	19.146 a	10.648 a
pH1	4.4500 a	400.71 a	138.52 a	5.5184 a	75.690 a	35.544 a	3.6944 a	94.38 b	16.229 a	8.840 a
pH2	4.0222 a	325.19 b	100.72 b	5.3963 a	75.690 a	33.196 a	3.5233 a	111.49 a	16.319 a	9.949 a
pH3	3.7500 a	293.61 b	97.69 b	5.3823 a	72.906 a	27.844 b	3.6944 a	83.72 c	15.548 a	10.351 a
Fe1	4.2556 a	357.86 a	125.07 a	5.4189 a	74.158 a	32.511 a	3.6011 a	82.30 c	16.079 a	8.521 a
Fe2	3.7889 a	318.66 a	99.56 a	5.4438 a	74.741 a	32.667 a	3.6167 a	99.29 b	15.355 a	8.713 a
Fe3	4.1778 a	343.00 a	112.31 a	5.4344 a	74.819 a	31.407 a	3.6322 a	108.00 a	16.662 a	11.906 a

Within a column means followed by the same letter are not significantly different at P<5% according to least significant different test

Table S6. The main effect of experimental treatment (AMF, pH and Fe) on biochemical characteristics and photosynthetic index of cucumber

Treatment	Fe conc. ($mg.kg^{-1}$ DW)	ABA ($mg^{-1}g$ FW)	APX (u/mg Pr)	SOD (u/mg Pr)	POX (u/mg Pr)	CAT (u/mg Pr)	Chlorophyll index (SPAD value)	Photosynthesis rate (µmol (CO_2) m^2s^{-1})	Transpiration (mmol(H_2O)m^2s^{-1})	Stomata Conductance (mmol (H_2O) m^2 s^{-1})	Mesophyll conductance (µmol)	Photosynthetic water use efficiency (µM mol CO_2 mol^{-1} H_2O)
AMF1	2.9249 a	97.028 a	0.6717 a	0.4558 a	0.7117 a	0.4171 a	22.417 b	4.9119 b	6.3637 b	0.2167 b	0.1967 a	34.516 b
AMF2	2.7622 a	85.842 b	0.5384 b	0.3709 b	0.5749 b	0.3357 b	28.222 a	6.5441 a	7.7074 a	0.2581 a	0.0289 b	40.023 a
pH1	2.7703 a	99.238 a	0.5945 b	0.4256 a	0.6300 b	0.3916 a	28.267 a	6.5467 a	6.2911 c	0.1994 c	0.2939 a	50.315 a
pH2	3.0179 a	82.683 b	0.5405 b	0.3712 b	0.5772 b	0.3356 b	27.686 a	5.8411 b	7.6333 a	0.2611 a	0.0311 b	34.010 b
pH3	2.7424 a	92.384 a	0.6801 a	0.4433 a	0.7229 a	0.4020 a	20.006 b	4.7961 c	7.1822 b	0.2517 b	0.0133 c	27.483 c
Fe1	1.2423 a	91.422 ab	0.5674 b	0.3921 b	0.6006 b	0.3602 b	22.036 b	4.8967 c	5.2600 c	0.1956 c	0.0294 b	34.012 c
Fe2	3.9369 a	97.985 a	0.6566 a	0.4386 a	0.6977 a	0.3989 a	25.138 ab	6.1122 b	7.4267 b	0.2422 b	0.0183 c	38.916 a
Fe3	3.3514 a	84.898 b	0.5912 b	0.4094 b	0.6317 ab	0.3701 b	28.784 a	6.1750 a	8.4200 a	0.2744 a	0.2906 a	38.881 b

Within a column means followed by the same letter are not significantly different at P<5% according to least significant different test

Table S7. The main effect of experimental treatment (AMF, pH and Fe) on stress index of cucumber

Treatment	TIFD	GMP	MP	HM	YSI	RI	SNPI	RMI
AMF1	1.6470 a	0.0337 a	0.3107 a	0.3033 a	1.6470 a	1.6470 a	0.5363 a	1.4526 a
AMF2	1.6578 a	0.0344 a	0.3196 a	0.3089 a	1.6578 a	1.6578 a	0.5448 a	1.4830 a
pH1	1.7811 a	0.0361 a	0.3217 a	0.3089 b	1.7811 a	1.7811 a	0.5728 a	1.4689 ab
pH2	1.8328 a	0.0383 a	0.3444 a	0.3361 a	1.8328 a	1.8328 a	0.5956 a	1.5983 a
pH3	1.3433 b	0.0278 b	0.2794 b	0.2733 c	1.3433 b	1.3433 b	0.4533 b	1.3361 b
Fe1	1.3789 b	0.0294 b	0.2650 c	0.2589 c	1.3789 b	1.3789 b	0.4506 c	1.2156 b
Fe2	1.6678 a	0.0339 ab	0.3161 b	0.3072 b	1.6678 a	1.6678 a	0.5439 b	1.4989 a
Fe3	1.9106 a	0.0389 a	0.3644 a	0.3522 a	1.9106 a	1.9106 a	0.6272 a	1.6889 a

Within a column means followed by the same letter are not significantly different at P<5% according to least significant different test

Table S8. The main effect of experimental treatment (AMF, pH and Fe) on Network root characteristics of cucumber

Treatment	Average Root Width (Diameter) (mm)	Network Depth	Network Length Distribution	Median Number of Roots	Network Area	Network Surface Area	Network Volume	Network Width to Depth Ratio
AMF1	390.70 a	1.0100 a	7518.3 a	310.08 a	0.0146 b	0.0041 b	0.0024 b	4924. 7 a
AMF2	337.01 b	1.1463 a	5746.3 a	211.98 b	0.3637 a	0.3573 a	0.3564 a	3550.5 a
pH1	369.31 a	1.1704 a	7632.9 a	276.66 a	0.1847 a	0.1748 a	0.1734 a	4501.9 a
pH2	395.25 a	1.0528 a	5126.6 a	261.36 a	0.1858 a	0.1773 a	0.1761 a	4620.8 a
pH3	327.01 b	1.0112 a	7137.5 a	245.08 a	0.1969 a	0.1901 a	0.1887 a	3590.1 a
Fe1	203.24 c	1.0054 a	3379.8 a	127.59 c	0.5378 a	0.5339 a	0.5334 a	2027.9 b
Fe2	399.97 b	1.0957 a	8317.6 a	279.33 b	0.0148 b	0.0039 b	0.0023 b	4453.5 a
Fe3	488.36 a	1.1333 a	8199.5 a	376.18 a	0.0148 b	0.0043 b	0.0024 b	6231.3 a

Within a column means followed by the same letter are not significantly different at P<5% according to least significant different test

In this study, significant effects of AMF inoculation, pH levels, and iron treatments on cucumber root characteristics were observed. The AMF1 treatment resulted in a significantly larger average root width (390.70 mm) and a greater median number of roots (310.08) compared to AMF2. Among the pH treatments, pH2 showed the widest root diameter (395.25 mm), while pH3 had the smallest (327.01 mm). Fe3 treatment (iron in amino chelate form) led to the most substantial improvements, with the largest average root width (488.36 mm), the highest median number of roots (376.18), and one of the greatest network length distributions (8199.5). In contrast, Fe1 treatment resulted in the smallest average root width (203.24 mm) and the lowest median number of roots (127.59). Additionally, Fe3 showed the highest network width to depth ratio (6231.3), indicating enhanced root development compared to other treatments.
